# Learning to play a musical instrument in the middle school is associated with superior audiovisual working memory and fluid intelligence: A cross-sectional behavioral study

**DOI:** 10.3389/fpsyg.2022.982704

**Published:** 2022-10-13

**Authors:** Mariangela Lippolis, Daniel Müllensiefen, Klaus Frieler, Benedetta Matarrelli, Peter Vuust, Rosalinda Cassibba, Elvira Brattico

**Affiliations:** ^1^Department of Teaching of Musical, Visual and Corporal Expression, University of Valencia, Valencia, Spain; ^2^Department of Psychology, Goldsmiths, University of London, London, United Kingdom; ^3^Department of Methodology, Max Planck Institute for Empirical Aesthetics, Frankfurt, Germany; ^4^Department of Clinical Medicine, Center for Music in the Brain (MIB), The Royal Academy of Music Aarhus and Aalborg, Aarhus University, Aarhus, Denmark; ^5^Department of Education, Psychology, and Communication, University of Bari Aldo Moro, Bari, Italy

**Keywords:** music training, cognitive development, audiovisual working memory, musical abilities, music education

## Abstract

Music training, in all its forms, is known to have an impact on behavior both in childhood and even in aging. In the delicate life period of transition from childhood to adulthood, music training might have a special role for behavioral and cognitive maturation. Among the several kinds of music training programs implemented in the educational communities, we focused on instrumental training incorporated in the public middle school curriculum in Italy that includes both individual, group and collective (orchestral) lessons several times a week. At three middle schools, we tested 285 preadolescent children (aged 10–14 years) with a test and questionnaire battery including adaptive tests for visuo-spatial working memory skills (with the Jack and Jill test), fluid intelligence (with a matrix reasoning test) and music-related perceptual and memory abilities (with listening tests). Of these children, 163 belonged to a music curriculum within the school and 122 to a standard curriculum. Significant differences between students of the music and standard curricula were found in both perceptual and cognitive domains, even when controlling for pre-existing individual differences in musical sophistication. The music children attending the third and last grade of middle school had better performance and showed the largest advantage compared to the control group on both audiovisual working memory and fluid intelligence. Furthermore, some gender differences were found for several tests and across groups in favor of females. The present results indicate that learning to play a musical instrument as part of the middle school curriculum represents a resource for preadolescent education. Even though the current evidence is not sufficient to establish the causality of the found effects, it can still guide future research evaluation with longitudinal data.

## Introduction

In recent years, several studies with continuous variables (e.g., duration of music training) have been carried out exhibiting the relationship between music training and overall cognitive ability both in childhood and in adulthood, making this area one of the most investigated within the field of music psychology (Schellenberg, 2006; Wetter et al., 2009; Degé et al., 2011; Corrigall et al., 2013; Swaminathan et al., 2017). Previous studies focusing on children (Schellenberg, 2004, 2006, 2011a) confirmed the hypothesis of a positive correlation of intelligence with the duration of musical courses, while reporting a long-term association between formal exposure to music in childhood and both IQ and academic performance.

Some studies showed a stronger relation of cognitive abilities with music lessons than with other leisure activities: in an intervention trial, participants allocated to music lessons showed greater increases in full-scale IQ points than students assigned to theater education or to a passive control group (Schellenberg, 2004). In a longitudinal study by Degé et al. (2011) with preschoolers, participants who took part in the phonological skills program or the music program significantly improved their phonological awareness, but this improvement was not seen in the sports group. Moreover, in some studies music training is associated with improvements in cognitive skills (Schlaug et al., 2005; Sachs et al., 2017; Guo et al., 2022). It is know that individuals with more music training have a significant advantage in tasks that require perceiving and discriminating between pitches, rhythms, and melodies (Wallentin et al., 2010; Law and Zentner, 2012; Zarate et al., 2012; Ireland et al., 2018). This means that a substantial correlation exists between music training and the cognitive abilities known as “near abilities,” which are promoted in music lessons; on the contrary, the concept of “far abilities” implies the transfer of the learner’s knowledge and skills from the taught context to another dissimilar context.

Correlational research revealed that individual differences in music lesson duration were associated with various “far” intellectual domains (Schellenberg, 2006). Music training involves procedural memory and long-term memory and, as it requires hours of practice, it includes the integration of auditory and visual information in a temporal framework; a study by Bugos and Mostafa (2011) showed a positive correlation between music training and information processing speed in the visual and auditory domains in adolescents with years of active music training compared to non-musicians. Further analyses on the relationship between music and cognitive abilities confirmed that children who receive music training have better performances in visuo-spatial abilities, language processing, verbal memory and reading ability (Jaschke et al., 2018; Linnavalli et al., 2018; James et al., 2020). In another study by Swaminathan and Schellenberg (2020), musical ability predicted language ability. Also, it has been found that learning to play an instrument as a child may predict academic performance and IQ in adulthood (Okada and Slevc, 2018).

Finding associations between music training and mental abilities like linguistic aptitude, visuo-spatial thinking, fluid reasoning and processing speed is fascinating and poses challenging issues concerning the underlying causes and developmental mechanisms. However, although there are favorable correlations between music training and cognitive skills, most of the relevant evidence is based on correlational studies, i.e., observational data, excluding causal inferences or only making it possible under certain conditions (Silas et al., 2022). Consequently, the very idea of non-musical benefits of music training is still questioned and debated in the scientific community.

According to some authors, the cause of improvements in cognitive abilities cannot be attributed solely to music training since transfer between very far abilities is, in human cognition, a rare effect (Gordon et al., 2015; Sala and Gobet, 2017, 2020). Moreover, most research on the relationship between learning music and improving cognitive ability is correlational, thus looking at whether continuous variables increase or decrease together. Hence, inference of causal effects from correlational studies can be confounded by pre-existing individual differences in social background, cognitive ability, personality, and genetic repertoire (Schellenberg, 2019; Vincenzi et al., 2022). Further issues, such as small sample size and no random assignment, together with the lack of an active control group, preclude the possibility of clearly determining causation between music training and non-musical abilities (Tierney and Kraus, 2013; McPherson, 2016; Schellenberg, 2020a).

In the study by Swaminathan and Schellenberg (2020), the link between music and language appeared to arise primarily from pre-existing factors and not from formal training in music. Moreover, literature showing brain structural differences between musicians and non-musicians has not yet ultimately established whether such differences result from pre-existing traits, musical training, or an interaction between the two (Norton et al., 2005; Vollmann et al., 2014; Olszewska et al., 2021). Hence, these findings impede one to make any definite inferences of causality in relation to cognitive transfer of music training. However, compelling proof of a causal link between musical experience and cognitive abilities comes from longitudinal experimental investigations.

The mentioned study by Degé et al. (2011), together with a study by Roden et al. (2014b) where results showed better performance in processing speed tasks in a group of children who followed music training for 1 year compared to children who followed a science training, are examples of longitudinal studies with active control groups. Even longitudinal studies with random assignment obtained enhanced reading skills and phonological awareness in musicians compared with controls (Cogo-Moreira et al., 2013; Slater et al., 2014; Rautenberg, 2015); this latter type of studies is actually the most effective way to address the issues regarding correlational studies (Tierney and Kraus, 2013). This also applies to the mentioned correlational literature investigating the relationship between music training and intelligence, unable to provide firm evidence on the effects of music training. Although, more recent studies on this topic suggest that the link between music training and IQ has an inverse causal relationship: children with higher IQ are rather more inclined to take music lessons than children with lower IQ (Schellenberg, 2019, 2020b; Kragness et al., 2021).

To achieve an “intelligent”, goal-oriented behavior (Arffa, 2007), individuals must be able to control their attention, inhibit their impulses, automatic responses and emotions, focus, maintain and protect attention from distraction, generate information and manipulate it in memory making it possible for us to engage in complex cognitive tasks, and adapt readily to changing circumstances. All these abilities correspond to the psychological concept of executive functions (EF; Arffa, 2007). In some studies, IQ has been found to be associated with executive functions although this remains a controversial topic (Friedman et al., 2006; Garcia-Molina et al., 2010; Ardila, 2018). Authors found some overlap between IQ and EFs. Specifically, EF refers to a group of skills that are often subdivided into the components of cognitive flexibility, working memory (WM) and inhibitory control (Stuss, 2011; Diamond, 2013; Marzocchi et al., 2020). The cognitive flexibility component comprises fluency, the ability to relinquish inhibition and generate information from mind and set shifting, also known as task switching, the ability to quickly shift between two activities or quickly switch response methods within a task. WM, according to the original definition by Baddeley and Hitch (1974), includes the ability to temporarily hold information in mind (maintenance) and manipulate it (updating), which relies on several interconnected operations: flexibility, mental manipulation and inhibition of distractors including visuo-spatial skills (Baddeley, 1986, 2000). Finally, inhibitory control is the suppression of inputs and behavioral reactions that are unrelated to the aim. Current developmental classifications distinguish between the ability to suppress interference from distracting stimuli (attentional inhibition) and the ability to block a preeminent motor response (response inhibition) (Tiego et al., 2018).

Each component of EF is known to mature at a distinct age and to develop at its own rate during childhood and adolescence; cognitive flexibility prolongs its development into young adulthood and, together with its subcomponents (set shifting and task switching), is the last to develop. This applies also to WM and inhibitory control which become distinct cognitive functions only after 9–10 years of age, although the basilar characteristics of both first appear about 6 months of age (Mata et al., 2017; Camos and Barrouillet, 2018; Tiego et al., 2018). The prolonged development of WM throughout adolescence is linked to brain changes, notably the prefrontal cortex’s maturation (Ferguson et al., 2021). Such rapid changes lead to an imbalance between the cognitive control system, relying on prefrontal brain structures and supporting the decision-making process through impulse inhibition, and the socioemotional system, relying on ventral striatum and orbitofrontal cortex (Donati et al., 2014; Meisel et al., 2019). This makes adolescents prone to risky behavioral decisions, and therefore it is important at this stage to possess a developed self-control. Being able to intentionally suppress or control behavioral reactions incompatible with one’s goals, is a sign of good self-regulation (Hofmann et al., 2012). As seen, inhibitory control and WM are two related but separate constructs; also, WM is supported by the same neural networks as cognitive flexibility which in turn relies on mental processes analogous to fluid intelligence (Stevens et al., 2002; Lambek and Shevlin, 2011; Barbey et al., 2014).

Fluid intelligence has been conceptualized as a discrete factor of general intelligence related to problem-solving and in contrast to crystallized intelligence which comes through learning (Cattell, 1963; Kent, 2017). Spearman’s (1927) theory hypothesized the presence of a universal factor that influences all cognitive processes called general intelligence (g); thus, g and lower-order skills like fluid intelligence are also correlated with auditory discrimination skills, since they share general and specialized genetic factors (Mosing et al., 2014; Silvia et al., 2016).

Notably, WM and fluid intelligence are closely associated functions and complementary measures of cognitive processes supporting complex cognition (Unsworth et al., 2014). WM capacity, allowing humans to flexibly create task-relevant connections between disparate bits of knowledge, hence, represents a key cognitive process underlying fluid intelligence together with focus of attention (Shipstead et al., 2016; Gray et al., 2017). Moreover, both fluid intelligence and WM are crucial for an adequate healthy development. On one hand, fluid intelligence allows for handling new problems without having prior knowledge of the task, and it is predictive of outcomes like educational success; also, it was found to be strongly associated with logical problem-solving and social adaptation (Huepe et al., 2011; Fuhrmann et al., 2020). On the other hand, disorders in WM functional maturation are linked to the emergence of neurodevelopmental disorders (Andre et al., 2016). The importance of a good cognitive and executive functioning in preadolescence and adolescence is, therefore, unquestioned; indeed, from childhood on, self-regulation is an important predictor of academic achievement and mental health (Woodward et al., 2017).

Due to the relatively gradual maturity of EFs, their course of development may be influenced by early activities through a variety of programs (Blair et al., 2008) including music training. Some of the most striking examples in the literature highlight the positive effect of music training on both neural and behavioral development: previous studies have shown that extra-curricular music training helps improve EF in children (Putkinen et al., 2015; Sachs et al., 2017; Shen et al., 2019; Frischen et al., 2021). Also, event-related potential and neuroimaging studies found evidence for improved EFs in musically trained children and adolescents (Zuk et al., 2014; Moreno et al., 2015; Kausel et al., 2020; Putkinen et al., 2021; Saarikivi, 2022) in addition to a more efficient use of neural systems supporting those functions (Putkinen and Saarikivi, 2018). Furthermore, in a longitudinal study of children from underprivileged backgrounds, those who took music lessons after school exhibited an enhanced ability to delay gratification compared to their counterparts who took sports or no after-school program; the music group also improved from 2 to 3 years on a test of response inhibition (Hennessy et al., 2019). In another study, Okada and Slevc (2018) found that actively participating in a music course requires the ability to consistently manipulate, remove and add information. In two recent experiments carried out by Chen et al. (2022), the level of music training was found to be positively correlated with response inhibition and WM abilities, implying that music training is linked to improved EF abilities in childhood. These studies assume that the intensity and length of practice are related to the degree of structural and functional adaptation in the brain (Habibi et al., 2018).

Some other studies focusing on the effects of musical education in childhood assume that music lessons can improve EFs, hence, boosting general cognitive advantages (Schellenberg and Peretz, 2008; Degé et al., 2011; Schellenberg and Winner, 2011; Holochwost et al., 2017). However, it remains unclear whether EFs play a role in the link between music lessons and general cognitive performance. In some cases, for example, the association between music training and IQ appears to be completely mediated by EFs while, in other cases, music training is correlated with IQ but not with EFs, except for WM (Swaminathan and Schellenberg, 2016). The effects on cognitive development may be influenced by the onset of music lessons tied to specific developmental stages and by a number of other relevant variables such as motivation, reward and contextual social factors.

In the specific case of WM, retention, processing and integration of complicated pitch and time sequences are all required in music training; consequently, music training and WM are clearly able to activate analogous mechanisms (Yurgil et al., 2020). When playing an instrument, WM integrates sound events, retrieves data from memory systems, connects sounds to meaning and memories, and aids in the development of emotional responses (Burunat et al., 2018; Criscuolo et al., 2019). The involvement of a WM network in musical tasks has been reported in several studies with higher brain activation in musicians than in non-musicians (Schulze et al., 2011b; Schulze and Koelsch, 2012; Burunat et al., 2014). Moreover, several positive effects on WM were found when comparing musicians and non-musicians. In previous studies, musicians outperformed non-musicians on *n*-back tasks showing that music training leads to improvements in WM tasks (Pallesen et al., 2010; Oechslin et al., 2013; Slevc et al., 2016; Ding et al., 2018). More recently, in Criscuolo et al. (2019) adult musicians showed higher general intelligence, verbal intelligence, WM and attention skills than non-musicians, while amateur musicians scored in between.

Despite this evidence, the findings of some studies of music training and WM in children and preadolescents are mixed. In a longitudinal study by Saarikivi et al. (2019), the results suggest that music training, particularly in late childhood, is significantly linked with improved WM capacity and maintenance but not with improved WM updating. Furthermore, some cross-sectional studies showed that musicians have an edge in auditory WM but not in visual WM (Strait et al., 2012). By contrast, in another study, Sachs et al. (2017) did not find any effect of music training on WM, although they did identify changes in neural activity in brain areas involved with cognitive functions between children who had received music training and those who had not (Sachs et al., 2017).

However, in a recent meta-analysis Lorenzen and Brattico (2020) provided a synthesized estimate of the effect of music training on a combined measure of short-term memory and WM in children, demonstrating that music training can increase these abilities. In another study by Bergman Nutley et al. (2014), the favorable effects of music practice on WM development, even controlling for parental education and other leisure activities, provide evidence for the significance of practice for WM growth during childhood and adolescence. Finally, in a study by Kausel et al. (2020), across attention conditions, preadolescent musicians performed better on bimodal (auditory/visual) attention tasks adapted by adding the memory retrieval task and showed, at the same time, more activity in fronto-parietal control network areas than controls.

Working memory is frequently studied separately for each modality, such as for visual and auditory WM. Auditory WM is the process of recalling sounds in mind for short periods of time when the sounds are no longer present in the environment; particularly, the expression “musical WM” specifically refers to the temporary storage and manipulation of musical inputs, like notations or musical sounds, useful for the completion of musical tasks (Silas et al., 2022). Auditory WM was also found to have a specific relationship with music sophistication (Lin et al., 2021; Lad et al., 2022; Silas et al., 2022), a multifaceted construct applicable to the general population since it focuses on multiple aspects of an individual’s musicality (improvisation, having a strong sense of pitch and rhythm, musical comprehension, appreciation, judgment, and communication abilities) which do not necessarily require mastering an instrument (Ollen, 2006; Müllensiefen et al., 2014; Zhang et al., 2020; Porflitt and Rosas, 2022). While several studies clearly indicate advantages of musicians in auditory WM (Schulze et al., 2011b; Kraus et al., 2012; Zuk et al., 2014; Slater and Kraus, 2016; Slevc et al., 2016; Alain et al., 2018; Guo et al., 2018; Nie et al., 2021), the evidence is controversial for visual WM.

The WM visuo-spatial sketchpad consists of two subsystems: a temporary visual store (“visual cache”) for visual shape and color, and a temporary spatial store (“inner scribe”) for movement sequences and planning (Logie, 1986, 2011). In a study by Hansen et al. (2013), significant effects were found in verbal WM but not in the visuo-spatial domain. These results converge with a more recent study involving adult participants where Talamini et al. (2017) found that musicians’ memory advantage is large for musical stimuli, medium for verbal stimuli (digits and words) and small for visual and/or spatial stimuli. Nevertheless, a particular relation has been found between music training and visuo-spatial sequence learning in the study by Anaya et al. (2017), showing that musicians may exhibit this domain enhanced due to fundamental differences in their visual-spatial abilities because of their extensive training and involvement in musical activities: encoding and recognition of visuo-temporal patterns is a common process during instrumental playing; think of reading a score or following the hands while playing (Janata and Grafton, 2003; Proverbio and Orlandi, 2016; Proverbio and Bellini, 2018). Also, a convergent finding by Rodrigues et al. (2014) suggests that musicians may score better on the visual memory test due to their enhanced sensorimotor connection. A further study conducted with children by Roden et al. (2014a) suggest an enhanced visual memory for the musicians, although without finding significant effect in those tests addressing the visuo-spatial sketchpad. In an additional longitudinal study with children, participants belonging to the music group exhibited significant improvements in the post-test compared to the sport group participants in visuo-spatial WM; however, no significant group x time interactions were found (Frischen et al., 2019).

As seen, previous research has been inconsistent on whether musical aptitude affects performance on complex non-musical tasks, such as tests of WM and intelligence, especially in preadolescence; thus, more investigation is needed on the topic. In the present study, we chose to focus on the two interrelated cognitive constructs of WM and fluid intelligence because they are central for the development of self-regulation abilities during preadolescence, preparing for adolescence and adulthood. Preadolescence is a period characterized by specific evolutionary tasks and milestones necessary for development, and the inattention to needs in this age range can have consequences for the functioning of adolescents and adults (Moore and Halle, 2001; Moore and Theokas, 2008; Fasano et al., 2019a). Hence, preadolescence can be thought of as a pivotal stage to understand the development of WM and its role together with fluid intelligence in academic achievement and psychosocial adaptation and, thus, a sensitive period for prevention.

### Instrumental education in music middle schools

In the educational communities, various forms of music training exist, and initial evidence suggests that even the type of training might affect transfer outcomes, with formal instrumental music training being more efficient than less structured programs (James et al., 2020). Hence, the complexity of musical development as well as the type of music training received need to be taken into account (Ilari et al., 2016). The present study focuses on a music training program carried out in Italian music middle schools, whose impact has only been little examined. Musical middle schools can be considered one of the largest and most original investments of the Italian public school, allowing tens of thousands of students to study a musical instrument for free within the curricular study pathway. The program was born in 1975 in some Italian middle schools following the traditional didactic model of the Conservatory until they became regulated starting from the academic year 2000/01. Characteristic elements of the regulation process were:

1) Emancipation from the traditional Conservatory teaching and the expansion of the range of possible activities: from individual lessons to group lessons, to the practice of ensemble music, to increasingly original forms of learning musical notation.
2) A strong link with curricular music education and with the didactic and educational programming of schools.
3) The ordinary and extended use of organizational and didactic autonomy for all schools.
4) The presence in schools of teachers having a Conservatory Master’s degree and specialized in the arrangement/composition of pieces suitable for students in that age group.

In musical middle schools it is possible for students to be enrolled in the standard curriculum as well: in fact, students enrolled in the music curriculum and in the standard curriculum attend their courses within the same school building. Both groups of students receive the same number and type of lessons in the morning; in the afternoon, only students enrolled in the music curriculum attend the instrument lessons two or three times a week. In some cases, separate course sections for students of the musical curriculum on one side and standard curriculum on the other side are set up within the same school building; otherwise, students of the music and standard curriculum are classmates. The morning lessons’ contents comply with the directives active throughout the national territory of the Italian Ministry of Education for lower secondary schools and include Italian, English and second community language, History, Geography, Mathematics, Science, Music, Art, Physical Education and Technology. Recently, the teaching of Citizenship and Constitution was added to these disciplines.^1^ In addition to the standard curriculum, numerous extra projects are offered, within the PON (National Operational Plan of the Italian Ministry of Education, funded by the European Social Fund and the European Regional Development Fund)^2^ to all students during the school year for creating an additional high quality, effective and fair education and training system. PON projects range from courses for strengthening basic school subjects to foreign language courses, computer labs, sport and artistic programs. One PON learning module lasts 30 h and, if necessary, can be repeated several times. Furthermore, these kinds of activities are also carried out during the summer months thanks to the “Summer Plan” of the Italian Ministry of Public Education.

Admission to the musical curriculum at the first middle school grade takes place through music aptitude tests at the beginning of the school year. Tests are regulated by law and consist of several steps: rhythmic and melodic abilities are evaluated through imitation tests where the candidate has to reproduce some musical phrases proposed by the teacher. Then, a pitch recognition test is followed by a first approach to the instruments (see footnote 1). After having tried several musical instruments, the candidate is asked to prioritize them to avoid the assignment of an instrument that is not particularly appreciated. Each student is assigned a numerical score by the examining board; it is necessary to obtain a minimum score in order to be admitted to the musical curriculum. At the end of the aptitude tests, a ranking of the admitted students is drawn up. Music middle schools curriculum, regulated by law by the Italian Ministry of Public Education, includes 2 h per week in the afternoon for 1st grade students and 3 h per week for 2nd and 3rd grade students. Classes are held in the afternoon and are organized as follows: 1 h of individual instrument class and/or in small groups; 1 h of collective class of music theory, music reading and group music; 1 h of group music class (orchestra and instrumental ensemble). Concerts are also performed during the school year.

Thus far, only one behavioral longitudinal study (Carioti et al., 2019) has been conducted on the peculiar kind of music instrument training offered at the Italian music middle schools, namely at the Negri-Calasanzio Middle School of Milan, involving a sample of 128 pre-adolescents: 72 students belonged to the music curriculum (30 with previous music experience and 42 without), and 56 belonged to the standard curriculum (44 with prior music experience and 12 without). Group differences in both musical and auditory cognitive skills were found, as well as in language processing and general cognitive abilities. Moreover, the music children showed superiority of memory, visuo-spatial, numerical and reading skills than the non-music children. However, no significant curriculum-by-time interactions were found. The authors, hence, concluded that pre-existing group differences might have a strong role in the observed behavioral findings; however, the study does not exclude that the school environment may bring benefits, and this needs to be investigated in future studies.

Altogether, we believe that the music middle school is a relevant context for investigating transfer effects of music training for several reasons: First, it offers the possibility of investigating music training carried out as a curricular, and thus mandatory, publicly-funded activity, while so far research has focused on extracurricular music activities conducted on a voluntary (typically self-financed) basis. If, on the one hand, extra-curricular activities have an impact on intrinsic motivational engagement in music and academic achievement (Pitts, 2007; Metsäpelto and Pulkkinen, 2012; Guo et al., 2022), on the other hand, including the instrumental training in the school curriculum may mean an increase of accuracy when studying and of the number of hours spent in music practice, which, in turn, may likely affect other skills in a different way, as well as provide an initial strong extrinsic motivation due to the evaluation of the instrumental course as one of the curricular subjects. Moreover, curricular training can reach students with various socio-economic backgrounds since materials and musical instruments are often given on loan for use to less well-off children.

Furthermore, studying music middle schools allows us to investigate the effects of this peculiar music training on the preadolescent population, and also how the peculiar environment and the daily opportunities to carry out music-related activities, together with group activities and other leisure activities, can contribute to balance the evolutionary trajectories and to foster a healthy development up to adolescence. Indeed, while several researchers consider primary school as an optimal environment for using music training as an aid to cognitive maturation, middle school targets the preadolescence phase in which music increases in importance, and musical identities start forming.

### The present study

This study is part of the MiddleMusic project, which was set up with the aim of collecting observational data on cognitive, emotional and performative skills, as well as on the sense of self-efficacy and general quality of life. Apart from the specific interest in musical middle schools, a particular focus of this project is on the preadolescent period and on the importance of self-regulation at this stage of development. Thus, this study aims to test the effects of middle school music training on two crucial cognitive constructs related to self-regulation, namely WM and fluid intelligence in preadolescence.

Three schools from three very different metropolitan areas participated. One of them was located in the city center, while the remaining two were located in suburban municipalities. According to the latest data provided by Istat (Italian National Institute of Statistics)^3^ for the year 2020 and similarly to previous years, an important gap can be found in the Apulian province of Bari between the city center and most of the municipalities in terms of per capita income and economic vulnerability. Hence, differently from the previous study by Carioti et al. (2019) focusing on a single school, the social contexts are very different according to the location, and therefore the socio-cultural level of the participants is extremely varied. Some studies in the past already focused on the effect on academic achievement and on the increase of cognitive abilities in musically-trained children at risk or with socio-cultural disadvantage: a 3-year longitudinal study by Rauscher and Hinton (2011) was conducted to determine whether music lessons could improve mathematics test and visuo-spatial test scores of economically disadvantaged elementary school children. Children at risk as well as middle-class children were assigned to music lessons and compared to control groups (computer lessons or no lessons). The results showed better performance by the musically-trained children only at the end of the third year of the study in those participants who had started music training before the age of 7. Furthermore, two longitudinal studies by Slater et al. (2014) demonstrate how music training can provide auditory and cognitive enrichment, helping children in critical developmental phase to improve their literacy skills and proceed with their academic progress. Also, a few recent investigations focused on collective music training, such as El Sistema offered for children coming from under-privileged areas (Alemán et al., 2017; Fasano et al., 2019b,in press) and showed a significant improvement of inhibitory control in the music group that was not found in the control group. Moreover, those children who did not follow any intense music program had a significant increase of hyperactivity-impulsivity from the pre- to the post-test, while the music group did not show a significant difference (Fasano et al., 2019b). However, more studies are required to account for the diversity of music training programs to scientifically inform future music education policies.

In the current study, we chose to focus on visuo-spatial and auditory WM. We did not test inhibitory control, although EFs often share correlates, and WM and inhibition in particular are closely related; indeed, inhibition tends to cohere more with WM measures than with measures of other types of inhibition (Diamond, 2013). The visuo-spatial aspect is crucial for the proper functioning of different cognitive skills and consequently for academic achievement, especially in the case of students with attention problems and ADHD (Gropper and Tannock, 2009; Giofrè et al., 2013, 2018; Mawjee et al., 2015). The Jack and Jill WM test is aimed specifically to measure the component of the visuo-spatial sketchpad in WM. It demonstrated strong correlations with other well-known tests of WM (Backward Digit Span, Memory Updating Figural, Corsi Block-tapping test), spatial tasks (Paper Folding, Shape Rotation, Mechanical Reasoning, Pattern Assembly), and a non-verbal intelligence test (Raven’s Progressive Matrices) (Tsigeman et al., 2022). This correlation is important as these tests are among the most used so far in the studies of WM and music (Talamini et al., 2017; Lorenzen and Brattico, 2020). Moreover, in this MiddleMusic project we used performance tests that were both used to measure music perception skills and musical sophistication (along with extensive questionnaires), but also served to assess auditory WM skills (Mas-Herrero et al., 2013, 2021; Müllensiefen et al., 2014). Hence, in addition to visuo-spatial skills we aimed to test auditory WM to provide a complete picture of the individual abilities that allow preadolescent children to explore multimodal world environments, in line with the increasing interest of researchers in multimodal cognition (Setti et al., 2021, 2022; Xie et al., 2021). The second scope of the study was to identify training-related changes in fluid intelligence, as assessed with a test based on Raven’s Progressive Matrices, currently one of the most accurate tests to evaluate both the ability to infer abstract relations and the ability to handle a broad range of problem-solving tasks in a dynamic way, which are the distinctive features of the g factor (Jensen, 1998; Holyoak, 2012; Martinez, 2013). In fact, the tests of fluid intelligence, which include creative problem-solving, are often the best ways to evaluate g (Carroll, 1993).

Moreover, we decided to investigate the gender variable. Examining gender variations in cognitive processes has garnered increasing attention in recent publications; particularly, the idea that each gender processes information using distinct cognitive strategies seems to be widely spread. According to prior literature, women would be inclined to elaborate information material in more depth, whereas males are more likely to be motivated by schemas or overarching information themes (Guillem and Mograss, 2005; Seinstra et al., 2015). In addition, females have been found to outperform males in word recalling, language and memory tests (Kaushanskaya et al., 2013; Loprinzi and Frith, 2018; Theofilidis et al., 2020) while males generally showed better performance in spatial abilities, especially in mental rotation (Levine et al., 2016; Lauer et al., 2019). However, the results are not always clear-cut, and they are often more related to the socio-cultural context and to the type of task (Wai et al., 2010; Nazareth et al., 2013; Miller and Halpern, 2014). Mostly, it was shown that the promotion or inhibition of cognitive abilities, such as spatial or verbal abilities, is highly mediated by gender-role. Boys and girls are encouraged by parents to engage in either stereotypically masculine or feminine activities appropriate to their gender, as “masculine” typed activities promote the development of spatial abilities while “feminine” typed activities pursuits instead strengthen of reading and language abilities (Reilly and Neumann, 2013). Despite all the evidence on gender differences in cognitive abilities, the relationship between EF performance in musicians compared to non-musicians and potential gender disparities have not yet been systematically studied (but see Jansen et al., 2022). Hence, it is pivotal to determine whether gender, especially in preadolescent age, can be a primary factor in the performance of cognitive tests.

The present study has a quasi-experimental research design, where cross-sectional analyses have been carried out. Moreover, the MiddleMusic project is still ongoing, and longitudinal data will be collected from the same children over the coming years. From the literature on the topic, it can be deduced that higher levels of musical ability are favorable for cognition performance, and according to previous studies musical ability can explain part of some cognitive performance factors, such as WM and fluid intelligence (Silvia et al., 2016; Slevc et al., 2016; Baker et al., 2018). As suggested by correlational evidence, we expected a replication of the association between following a music training program within the school curriculum and the performance on tests of cognitive and musical abilities (possibly due to pre-existing differences and selection). We also expected older students to perform better on the cognitive and musical tests (due to maturation). Moreover, we expected to find a stronger growth in abilities for the music group than for the control group across age groups (due to the effects of music training). Overall, we expected to find differences in favor of musicians beyond all the possible confounders and covariates. Finally, considering the literature on gender differences, we expected to find significantly better performance in females due to the developmental differences.

## Materials and methods

### Ethics

The present study is part of a larger project (“MiddleMusic”) which involves a longitudinal analysis with the same middle schools over an extended time period and involves parents through the administration of three questionnaires in paper format. In addition to the sample of children analyzed for the present study, samples of adult participants were included in the MiddleMusic project with the aim of carrying out the Italian validation of some instruments. The research protocol has been approved by the Ethics Committee of the Department of Education, Psychology and Communication of the University of Bari “Aldo Moro” (reference: ET-21-15).

### Participants

The sample includes 324 preadolescents (range 10–14 years; mean age = 12.3 SD = 0.94) selected from 21 classes of three music-focused middle schools in the Bari area (Apulia, Italy): De Amicis-Dizonno (Triggiano), Alighieri-Tanzi (Mola di Bari), and Massari-Galilei (Bari). Children (47.2% females and 52.8% males) belonged either to the musical curriculum (“musicians” *N* = 194) or to the standard curriculum (non-musicians *N* = 130).

The musicians were compared with their schoolmates or classmates enrolled in the standard curriculum. This was a necessary choice since in this kind of community-based studies choosing whether a child should participate in the music curriculum or the conventional curriculum at school is neither logistically possible nor ethically acceptable. For practical reasons especially due to COVID-19, we were unable to collect more specific information on prior musical expertise and general background of participants through preliminary interviews as in the case of the study conducted by Carioti et al. (2019). Instead, we administered extensive questionnaires (Gold-MSI, detailed below) in order to obtain information on their level of expertise, start age and amount of music training. Moreover, we aimed to evaluate the children’s musical background at the start of the study in order to determine the impact of music training on audiovisual WM and fluid intelligence.

The music training program of the music curriculum consists of 2 h of general musical lessons per week; the standard curriculum does not include additional afternoon lessons. For this reason, standard curriculum students tend to have more free time and have more chances to attend the extra above-mentioned PON projects within their own schools. In any case, the spending of free time should be investigated more in depth, as no data in this respect are available in the present study. Such projects were also running during the measurement periods and addressed also to the classes involved in the study. For example, within the “Summer Plan” 2022, among the projects presented by the schools, the most popular themes were the following: strengthening of functional alphabetic competence (19%) learning modules to enhance language skills (17%), strengthening of “STEM” subjects - Science, Technology, Engineering and Mathematical Subjects (14%), Digital Strengthening (10%), Physical Education (10%) art theater creative writing (9%), cultural awareness and expression (9%), music and singing (5%). And, moreover, in the general plan creative and artisanal workshops, education for active citizenship and education for legality and human rights projects are foreseen (see text footnote 1).

For example, some of the activities carried out in the last year at the three participating schools consisted in: entertaining logical puzzles (gamification), digital competence modules, “Edugreen” workshops on eco-sustainability, modules for the strengthening of English and French languages, creative writing and finally, multi-disciplinary group artistic workshops.^4^

Data were collected for the most part in December 2021; only a small group attending 2nd and 3rd years of middle school was tested in January 2022.

### Tests and questionnaires

The MiddleMusic project shares several objectives with the LongGold project: LongGold investigates and follows groups of secondary school students for several years in order to demonstrate how longitudinal research may give a solid empirical foundation for understanding the origins and impacts of musical engagement (Müllensiefen and Harrison, 2020). Students are assessed once a year and complete a test battery of musical and non-musical tasks as well as questionnaires asking about music and other free-time activities, self-concepts, personal strengths and difficulties, personality and other person-related aspects. The battery runs in a standard internet browser, and pupils are tested in their classroom. LongGold researchers use school computers or can supply tablets and headphones to perform the test sessions, depending on the school facilities.^5^

One of the main differences between the present study and the study by Carioti et al. (2019) as well as other studies is our use of fairly entertaining and motivating ability and performance tests to keep motivation high: in fact, tasks such as the Digit Span task often employed in this type of studies can be perceived as boring and repeating to children, and this could negatively affect the performance. All cognitive and performance tests use an adaptive procedure which homes in on the participant’s individual ability level as quickly as possible and therefore saves a lot of testing time. The adaptive testing methods and underlying statistical theory of the musical ability tests employed in this study are explained in Harrison et al. (2017), Harrison and Müllensiefen (2018), and Larrouy-Maestri et al. (2019). The method of computerized adaptive testing (CAT) involves selecting test items using an algorithm based on the test-taker’s previous replies. The goal of item selection is often to maximize the amount of information each item conveys regarding the test-taker’s actual aptitude. This gives a considerable advantage in improving the testing efficiency and the test reliability, as adaptive testing aims to give the most informative items possible at each testing point.

To administer the tests, we employed the psychTestR online testing framework (Harrison, 2020). Several adaptive ability tests have been developed in this framework, including the melody discrimination test (MDT; Harrison et al., 2017), the beat perception test (BAT; Harrison and Müllensiefen, 2018), the mistuning perception test (MPT; Larrouy-Maestri et al., 2019), the visuospatial WM test (JAJ; Tsigeman et al., 2022), and the rhythmic ability test (RAT; MacGregor et al., 2022). In this study we used a selection of tests and questionnaires as follows, summarized also in Table 1. To measure cognitive abilities the Jack and Jill and the Matrix Reasoning were used.

**TABLE 1:** LongGold tests employed in the MiddleMusic project.

Area	Test	Acronym	References
IQ	Matrix reasoning	MIQ	Condon and Revelle, 2014; Chan and Kosinski, 2015
Visuo-spatial WM	Jack&Jill Working Memory Test	JAJ	Tsigeman et al., 2022
Music abilities	Computerized Adaptive Beat Alignment Test	CA-BAT	Harrison and Müllensiefen, 2018
	Melody Discrimination Test	MDT	Harrison et al., 2017
	Mistuning Perception Test	MPT	Larrouy-Maestri et al., 2019
	Emotion Discrimination Test	EDT	MacGregor and Müllensiefen, 2019
	Rhythm Ability Test	RAT	MacGregor et al., 2022
Music Sophistication	Goldsmiths Musical Sophistication Index	Gold-MSI	Müllensiefen et al., 2014

The Jack&Jill WM test (JAJ) measures visuo-spatial WM capacity employing a dual task paradigm (Tsigeman et al., 2022). The task is adaptive and based on an explanatory item response theory (IRT) model. The final IRT scores of the test show a bell-shaped distribution that is centered around 0. Negative scores indicate a performance worse than the average of the calibration sample, and positive scores indicate a better than average performance. The test is implemented in the open-source R package JAJ.^6^ The test length on the LongGold project was set to eight trials. In the present study, the participants completed a total of 7 trials for the JAJ since in the original study the authors found that reliability and validity for this test are acceptable after 7 or 8 trials (5–6 mins), with diminishing gains from more trials (Tsigeman et al., 2022).

Many of the classic tests used for WM require keeping the information while working on a second task, which can cause interference, and the Jack and Jill test is also based on this dual-task paradigm. The JAJ task presents participants with pictures of a young female on the left (“Jill”) and a young male on the right (“Jack”) of the screen on a white background. Before the task, the participant is instructed on the procedures through a tutorial. The task is divided into trials of different lengths increasing over time, totaling 14 trials in one testing session.

While Jack rotates about his axis on each stimulus presentation and can grasp a ball in either of his right or left hand, Jill always remains in the same position while clutching a blue ball in her right hand. Additionally, Jack’s ball moves, randomly taking one of the six indicated spots on the screen (orange dots). Participants must complete two tasks for each stimulus presentation: (1) determining whether Jack is holding the ball in the same hand as Jill, and (2) memorizing the location of the ball at that moment. Participants are required to click on the designated spots in the proper order at the conclusion of each trial in order to recall the order of the ball positions.

Correct answers are represented by cursor positions. Responses from participants are evaluated as correct (1) or wrong (0) depending on whether the entirety of a trial can be successfully repeated. The duration of the sequence of ball placements that a participant can recall serves as an indicator of their visuo-spatial WM span. Because the JAJ task needs participants to alternate between memory encoding (memorizing the position of the ball) and mental rotation with a subsequent choice on visual input, which adds additional cognitive strain, it can be classified as a complex span task.

Matrix Reasoning (MIQ) is a test that contains stimuli that are similar to those used in Raven’s Progressive Matrices (Condon and Revelle, 2014; Chan and Kosinski, 2015). The stimuli are 3 × 3 arrays of geometric shapes with one of the nine shapes missing. Participants are instructed to identify which of the six geometric shapes presented as response choices will best complete the stimuli. In each round the stimulus disappears after 120 s, leaving only the response options visible for the participant.

The Computerized Adaptive Beat Alignment Test (CA-BAT) is a test that probes a listener’s beat perception ability using the beat alignment paradigm where the listener tries to detect misalignment between a metronome and a musical extract (Harrison and Müllensiefen, 2018). This paradigm comprises a series of two alternative forced choice trials. Each task presents the participant with two versions of a music track, both overlaid on a metronome-like beep track. In one version, the target beep track is exactly in time with the locations of the musical beats. In the other version, the beep track is “off” from the main musical beat. The participant’s task is to identify whether the track is “on” or “off” the beat. The CA-BAT is an adaptive test. Therefore, the item selection adapts to the candidate’s previous responses. Typically, the goal is to maximize the amount of information each subsequent item provides about the candidate’s true skill level, which in practice means that poor performers receive easier items and better performers receive more difficult items.

Melody Discrimination Test (MDT) is an adaptive test characterized by a three-alternative forced-choice (3-AFC) melodic discrimination paradigm (Harrison et al., 2017). In the 3-AFC paradigm, each melody version is compared with every other version, producing three similarity comparisons in total; in addition, item difficulty is determined by manipulating the melody’s length. The participant performs a decision-making process to determine which melody was the odd-one-out, on the basis of these similarity judgments. In each trial, the participant is presented with three versions of the same melody. Two of these versions have the same interval structure and are called lures, but one version has exactly one altered note and is called odd. These three versions can occur in any order, and the participant’s job is to identify which version is the odd one. Two of these melody pairs must be different, and one must be the same. The participant’s task is therefore to identify the most similar pair, and then the odd-one-out must be the melody not contained within this pair. The duration of the test may vary according to the specific needs of the research; in the case of our study, the test consisted of 18 items.

Mistuning Perception Test (MPT) is a test that uses a two-alternative forced choice (2-AFC) paradigm in which each trial comprises two versions of the same musical extract, one “in tune” and the other “out of tune” (Larrouy-Maestri et al., 2019). The stimulus material consists of short excerpts (6–12 s in length) from pop music performances (obtained from MedleyDB; Bittner et al., 2017) for which the vocal track was pitch-shifted relative to the instrumental tracks. Each musical extract has a vocalist singing the main melodic line and an instrumental accompaniment. Out-of-tune extracts are produced by adding a constant pitch shift to the vocal line. The listener’s task is then to identify which of the pair was the out-of-tune version.

Rhythm Ability Test (RAT) is an adaptive test in which participants have to select the correct image that corresponds to the rhythm heard (MacGregor et al., 2022). The rhythms are made up of high-pitched and low-pitched sounds. High-pitched and low-pitched sounds are represented in each picture with two rows of squares of two different colors placed at the top row (to indicate high-pitched sounds) and at the bottom row (to indicate low-pitched sounds). The tasks are presented according to a progressive increasing difficulty, and the stimuli are made up of 4, 8, and 16 sounds (quarter, eights, sixteenths notes).

Emotion Discrimination Test (EDT) is an adaptive test and uses a two-alternative forced choice (2-AFC) format: two played or sung fragments of the same melody are presented for each task (MacGregor and Müllensiefen, 2019). The participant’s task is to identify which of the two fragments corresponds to the expression of a specific emotion (anger, happiness, sadness, and tenderness). Excerpts differ between trials in terms of musical features such as length, instrument, melody, target emotion and comparison emotion, and item difficulty is assessed with regard to the contribution of these features. The key features of angry excerpts are high amplitude, fast tempo and greater roughness, happy excerpts are similar to angry excerpts though without high roughness, sad excerpts exhibit slow tempos and low amplitude, and tender excerpts have similar acoustic properties as those conveying sadness.

In addition to the cognitive and music perception tests, the pre-adolescent participants were asked to complete a demographic questionnaire and the Gold-MSI, a 39-item self-report scale that comprises five subscales (Active Musical Engagement, Perceptual Abilities, Music Training, Singing Abilities, and Emotional Engagement with Music) and one general factor (General Musical Sophistication) (Müllensiefen et al., 2014).

The Active Engagement factor includes a variety of active musical engagement behaviors, including the conscious devoting of time and money to musical activities; the Perceptual Abilities factor represents the self-assessment of various musical abilities, most of which are related to music listening skills; the music training factor concerns one’s level of self-assessed musicianship, as well as the amount of music training and practice; the Singing Abilities factor reflects different skills and activities related to singing; the Emotions factor covers different and mainly active behaviors related to emotional responses to music; and finally, the General Musical Sophistication factor incorporates aspects from all above mentioned five sub-scales.

Initially developed for use with adults, the factor structure and internal reliability of the Gold-MSI have been replicated and validated for the use with secondary school pupils in a large German sample of 11–19-year-olds (Schaal et al., 2014; Fiedler and Müllensiefen, 2015). All the perceptive test instructions were translated into Italian from English. The Gold-MSI was translated with the method of the back translation (similar to the method described in Lin et al., 2021): Two parallel translations were carried out and the two versions of the same test, translated from English to Italian and from Italian to English, were secondly compared with a native speaker.

### Procedures

The data collection took place in the schools’ IT rooms, and the whole process was guided by the experimenters. The tests were administered during class hours for a total duration of 2 h after prior agreements with principals and teachers and after having received informed consent signed by parents. The tests were administered on a specific online platform implemented by the LongGold group which can be accessed through a link (a demo version of the LongGold tests can be found here: https://longgold.org/tests/). In addition, unique numerical identification codes were created for each participant. The battery of tests was split into two parts and administered in two sessions on two different days to avoid cognitive overload and not to compromise the performance. The order of tasks was balanced by type and required level of cognitive load; the cognitive tasks were placed at the very beginning of the battery, the perceptual tests and questionnaires were presented alternately to keep the participant’s attention high.

The JAJ test was administered within the second session. Due to the non-attendance at school of a group of students on the second day session, the participants in the JAJ test appear to be fewer in number than the participants who completed the remaining tests. Of the children invited and who consented to participate, 29 were not at school due to COVID reasons on the second day of measurement, and 10 more missed to complete only the first part of the battery. For this reason, the final sample obtained includes 285 participants instead of 324 (47.7% females and 52.3% males) belonging to a music class (*N* = 163) or a class standard (*N* = 122).

As reported in Table 2, the sample of students involved was equally distributed among the three grades; in each group the age of the participants ranged between 10 and 14 years. In the original sample, 14 participants had learning disabilities or impairments, but they were not excluded from the present analysis as they presented mild cognitive disabilities. In the final sample, only 10 of them remained (six musicians and four non-musicians) and their scores appeared not to be dissimilar to those obtained by the rest of the participants. None of the participants reported a salient hearing impairment, and no differences were found in the scores of the only participant who declared to have an increased hearing sensitivity than normal. Finally, some data were missing for the Rhythm Ability Test (*n* = 1), the Mistuning Perception Test (*n* = 3) and the Start Age sub-scale of the Gold-MSI (*n* = 27) from non-musicians only.

**TABLE 2:** Sample descriptives.

		*N*	Mean age	SD	Gender	
					
					*F*	*M*
**1st Grade**						
Group	M	56	11.3	0.31	22	34
	NM	51	11.3	0.29	24	27
**2nd Grade**						
Group	M	50	12.3	0.30	29	21
	NM	30	12.2	0.37	12	18
**3rd Grade**						
Group	M	57	13.3	0.35	26	31
	NM	41	13.4	0.38	23	18

M, musicians; NM, non-musicians.

### Data analyses

Statistical analyses were performed using the Jamovi software (version 2.2, 2021, retrieved from https://www.jamovi.org/; R Core Team, 2021). As a first step, an independent sample *t*-test was performed to detect whether there were significant differences in general music sophistication in the 1st grade. Subsequently, a series of two-factor ANOVAs were performed to verify the two main basic hypotheses and to detect whether there were significant differences in cognitive and musical skills for age and group. Therefore, two-factor ANOVAs were performed using the two independent variables Grade (1/2/3) and Group (Musicians/Non-musicians) on the following dependent variables:

1. Working Memory (JAJ: Jack&Jill Test);
2. IQ (MIQ: Matrix Reasoning Test);
3. Beat Perception (BAT: Beat Alignment Perception Test);
4. Melody Discrimination (MDT: Melodic Discrimination Test);
5. Mistuning Perception (MPT: Mistuning Discrimination Test);
6. Emotion Discrimination (EDT: Emotion Discrimination Test);
7. Rhythmic Ability (RAT: Rhythm Ability Test).

Subsequently, gender was also included as a further independent variable in the analyses. For the same variables, a series of ANCOVAs were performed treating the Gold-MSI General Index score as a covariate to account for probable pre-existing differences, and also due to lack of subjects’ random assignment. Moreover, general musical sophistication was found to correlate with self-reported music training (Müllensiefen et al., 2014). As the students of the musical curriculum are chosen through aptitude tests based on imitation and recognition of rhythm, melody and pitch, we aimed to statistically control for pre-existing individual differences in musicality to better see the effect of music training, similarly to other studies where some Gold-MSI subscales were used as covariates (Kang and Williamson, 2012; Farrugia et al., 2015; Ma et al., 2021; Wallmark et al., 2021; Krause et al., 2022).

It is important to note that the Gold-MSI has a hierarchical factor structure, and the General value is the mean of an assorted set of items across all the other subscales. In the Gold-MSI original study by Müllensiefen et al. (2014), 18 items were drawn from all five Gold-MSI subscales to index musical sophistication in general, with a clear preponderance of items from the Musical Training and the Singing Abilities subscales. In addition, for removing outliers we applied a filter in the analysis of MIQ scores that was based on the estimated amount of measurement error, rejecting all observations with a standard error of the mean (SEM) value < 1.5 with the aim to eliminate performance scores which were estimated in the presence of a high amount of measurement error, thereby eliminating nine more participant’s scores from the dataset. For the JAJ no outliers were found.

## Results

The scores of the adaptive tests were given on a z-scale and had a population mean around 0 and ranged from −4 to 4. JAJ and MIQ score ranges, mean and standard deviations for each grade and group are summarized in Table 3, likewise the SEM ranges for both JAJ and MIQ.

**TABLE 3:** Jack&Jill (JAJ) and matrix reasoning (MIQ) descriptives for grades and groups and gender.

	Grade	Group	Gender	*N*	Mean	SD
JAJ	1	M	Female	22	−0.18621	0.950
			Male	32	−0.09296	0.912
		NM	Female	24	−0.29614	0.879
			Male	27	−0.32285	0.675
	2	M	Female	26	0.00176	0.828
			Male	21	0.18652	0.864
		NM	Female	12	−0.07302	0.684
			Male	18	0.13442	0.669
	3	M	Female	25	0.46949	0.714
			Male	29	0.04914	0.939
		NM	Female	22	−0.28995	0.827
			Male	18	−0.09424	0.853
MIQ	1	M	Female	22	−2.32072	0.767
			Male	32	−1.84395	0.936
		NM	Female	24	−2.08777	0.982
			Male	27	−2.48786	0.686
	2	M	Female	26	−1.87805	0.675
			Male	21	−1.65372	0.974
		NM	Female	12	−2.16634	0.914
			Male	18	−2.00237	0.799
	3	M	Female	25	−1.62141	0.895
			Male	29	−1.61453	0.887
		NM	Female	22	−2.08135	1.010
			Male	18	−2.10286	1.042

M, musicians; NM, non-musicians.

Overall, JAJ scores ranged [−1.80, 2.83] with a mean of 0.016 (SD = 0.89) while MIQ scores ranged [−3.59, 1.71] with a mean of −1.87 (SD = 0.93). For the JAJ, the lowest ability value (−1.89) would correspond to the ability of remembering not more than one position consistently (Tsigeman et al., 2022). Considering this, in the present study 47.4% of the participants obtained a score below zero for the JAJ: of them, 48.1% are musicians and 51.8 non-musicians. Moreover, the 21.7% of participants who obtained scores below 0 attend the 1st grade. Regarding gender, no significant differences were found.

A comparison of estimated marginal means showed the following: For the music group 1st Grade MIQ scores ranged [−2.31, −1.82] while for the 1st Grade non-musicians scores ranged [−2.58, −2.08]; 3rd Grades musicians scores ranged [−1.83, −1.34] and 3rd Grade non-musicians scores ranged [−2.42, −1.85]. For JAJ, 1st Grade musicians scores ranged [−0.35, 0.10] and 1st Grade non-musicians scores ranged [−0.58, −0.10]; 3rd Grade musicians scores ranged [0.06, 0.51] while 3rd Grade non-musicians scores ranged [−0.50, 0.03].

Comparing the estimated marginal means for the perceptive tests showed similar results. Descriptive statistics for all the perceptual tests can be also found in Table 4: BAT (*M* = −1; SD = 1.23); MDT (*M* = −1.14; SD = 1.08); MPT (*M* = −0.7; SD = 1.15); EDT (*M* = 0.6; SD = 0.9) RAT (*M* = 0.6; SD = 0.9). For all the three grades, higher mean scores can be always found for the musicians compared to non-musicians, and overall, both groups tend to improve their scores in 3rd grade compared to 1st.

**TABLE 4:** Descriptive statistics for all the perceptive tests.

	Grade	Grade	Gender	*N*	Mean	SD
CA-BAT	1	M	Female	22	−0.8484	1.062
			Male	32	−1.3194	1.149
		NM	Female	24	−1.5169	1.365
			Male	27	−1.6951	1.418
	2	M	Female	26	−0.4379	0.827
			Male	21	−0.6774	0.999
		NM	Female	12	−0.9597	1.005
			Male	18	−0.9679	1.339
	3	M	Female	25	−0.5237	1.164
			Male	29	−1.0052	1.336
		NM	Female	22	−1.0502	1.068
			Male	18	−1.4549	1.555
MDT	1	M	Female	22	−1.0957	1.104
			Male	32	−1.3840	0.917
		NM	Female	24	−1.6999	1.095
			Male	27	−1.4922	1.132
	2	M	Female	26	−0.9481	0.898
			Male	21	−1.0231	1.104
		NM	Female	12	−1.6880	0.762
			Male	18	−1.1849	1.111
	3	M	Female	25	−0.5490	1.069
			Male	29	−0.7132	1.050
		NM	Female	22	−1.2212	0.985
			Male	18	−1.5660	1.054
MPT	1	M	Female	22	−0.6690	1.056
			Male	32	−0.9477	1.045
		NM	Female	22	−1.1373	1.351
			Male	26	−1.6432	1.310
	2	M	Female	26	−0.2969	1.067
			Male	21	−0.7156	1.095
		NM	Female	12	−1.1736	1.182
			Male	18	−0.5925	1.188
	3	M	Female	25	−0.0897	0.775
			Male	29	−0.6441	1.132
		NM	Female	22	−0.2583	1.106
			Male	18	−1.2586	0.854
EDT	1	M	Female	22	0.5932	1.117
			Male	32	0.5068	0.859
		NM	Female	24	0.4786	0.999
			Male	27	0.1615	1.051
	2	M	Female	26	0.6833	0.948
			Male	21	0.8154	1.051
		NM	Female	12	0.9135	1.033
			Male	18	0.5330	1.013
	3	M	Female	25	1.1620	0.781
			Male	29	0.5642	0.997
		NM	Female	22	0.8740	0.961
			Male	18	0.4510	0.971
RAT	1	M	Female	22	−0.6754	0.923
			Male	32	−0.6418	0.949
		NM	Female	24	−1.0240	0.779
			Male	26	−1.1966	0.790
	2	M	Female	26	−0.7967	0.823
			Male	21	−0.3189	0.937
		NM	Female	12	−1.0394	0.811
			Male	18	−0.6794	0.850
	3	M	Female	25	−0.1251	0.757
			Male	29	−0.4099	0.910
		NM	Female	22	−0.9258	0.808
			Male	18	−0.8501	0.859

(1) M, musicians; NM, non-musicians.

(2) JAJ, Jack&Jill Working Memory Test; MIQ, matrix reasoning; CA-BAT, Computerized Adaptive Beat Alignment Test; MDT, Melody Discrimination Test; MPT, Mistuning Perception Test; EDT, Emotion Discrimination Test; RAT, Rhythm Ability Test.

Table 5 reports descriptive statistics for the GOLD-MSI. Musicians show better scores than non-musicians in all sub-scales except for Starting Age; however, the data missing from 27 non-musicians may have affected the results. Another exception was found in the sub-scale Emotion for the 2nd Grade, where musicians (*M* = 5.14; SD = 0.99) and non-musicians (*M* = 5.14; SD = 1.20) have the same mean scores.

**TABLE 5:** Gold-MSI descriptives for all subscales.

	Grade	Group	*N*	Missing	Mean	SD
GMSI	1	M	56	0	4.21	0.791
		NM	51	0	3.59	0.752
	2	M	50	0	4.40	0.858
		NM	30	0	3.89	0.918
	3	M	57	0	4.35	0.761
		NM	41	0	3.52	0.844
GMS active engagement	1	M	56	0	4.43	0.966
		NM	51	0	3.89	0.970
	2	M	50	0	4.71	1.012
		NM	30	0	4.62	1.130
	3	M	57	0	4.50	1.011
		NM	41	0	4.00	0.909
GMS music training	1	M	56	0	3.82	0.931
		NM	51	0	3.17	1.292
	2	M	50	0	4.25	0.898
		NM	30	0	3.25	1.203
	3	M	57	0	4.25	0.833
		NM	41	0	3.20	1.120
GMS emotions	1	M	56	0	4.57	0.957
		NM	51	0	4.36	1.122
	2	M	50	0	5.14	0.994
		NM	30	0	5.14	1.205
	3	M	57	0	5.09	1.047
		NM	41	0	4.80	0.979
GMS perceptual abilities	1	M	56	0	4.41	0.849
		NM	51	0	4.08	0.789
	2	M	50	0	4.81	0.919
		NM	30	0	4.43	0.776
	3	M	57	0	4.73	0.708
		NM	41	0	4.31	0.790
GMS singing abilities	1	M	56	0	4.71	1.081
		NM	51	0	4.27	1.014
	2	M	50	0	4.67	0.991
		NM	30	0	4.42	1.011
	3	M	57	0	4.52	0.763
		NM	41	0	3.89	1.071
GMS instrument	1	M	56	0	7.02	5.629
		NM	51	0	6.45	7.739
	2	M	50	0	7.54	5.736
		NM	30	0	5.77	6.135
	3	M	57	0	7.44	5.720
		NM	41	0	5.17	6.025
GMS start age	1	M	56	0	7.70	2.207
		NM	40	11	8.63	1.877
	2	M	50	0	7.42	1.939
		NM	25	5	7.36	2.447
	3	M	57	0	7.77	1.899
		NM	30	11	8.47	1.737

(1) M, musicians; NM, non-musicians.

(2) The number of participants is lower than the sample number as a filter was applied to SEM values <1.5 which removed the score of nine participants.

As we aimed to test whether there are pre-existing differences in musical sophistication and expertise between the two groups when they enter middle school, we decided to perform a *t*-test as the first step. The results of the independent sample *t*-test indeed showed a significant difference between the groups of participants enrolled in the 1st grade of middle school (Table 6).

**TABLE 6:** Independent samples *t*-test for 1st grade groups using general music sophistication as a dependent variable.

						95% confidence interval			
						
	Statistic	df	*p*	Difference in means	SE difference	Lower	Upper		Effect size
Student’s *t*	4.11	105	<0.001	0.588	0.143	0.304	0.873	Cohen’s *d*	0.795
Welch’s *t*	4.12	105	<0.001	0.588	0.143	0.305	0.872	Cohen’s *d*	0.796
Mann-Whitney *U*	777		<0.001	0.667		0.333	0.933	Rank biserial correlation	0.456

The 56 1st grade musicians (*M* = 4.28; SD = 0.765) compared to the 51 participants in the control group (*M* = 3.69; SD = 0.712) demonstrated better scores: *t*(105) = 4.11; *p* < 0.001; *d* = 0.795. Welch’s test results also suggest a significant difference between the two groups: *t*(105) = 4.12; *p* < 0.001. Also, Mann-Whitney test reported similar results: as the *p* value obtained from the Mann-Whitney *U* test is significant (*U* = 777, *p* < 0.001), we conclude that the general level of musical sophistication of the two groups is significantly different. Moreover, the general Gold-MSI score was found to be distributed normally, as verified by the Shapiro-Wilk test (*p* = 0.648).

**FIGURE 1:**
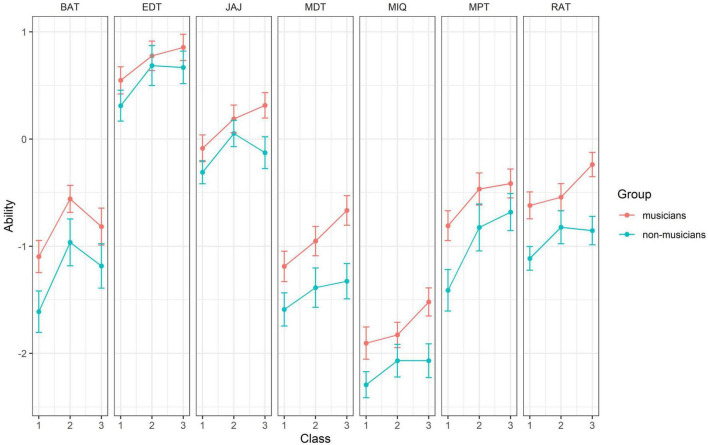
Mean ability values for the seven performance tests by grade and quasi-experimental group. Error bars indicate standard error of mean. BAT, Beat Alignment Test; EDT, Emotion Discrimination Test; JAJ, Jack&Jill Working Memory Test; MDT, Melody Discrimination Test; MIQ, matrix reasoning; MPT, Mistuning Perception Test; RAT, Rhythm Ability Test.

The subsequent series of 7 two-factor ANOVAs showed a significant difference for both of the cognitive tests, namely JAJ and MIQ, with higher scores for musicians than non-musicians (JAJ: df = 1; *F* = 6.101; *p* = 0.025; ηp^2^ = 0.018; MIQ: df = 1; *F* = 8.462; *p* = 0.004; ηp^2^ = 0.030). The analysis also showed a robust difference between the groups for both JAJ and MIQ especially in 3rd grade. This and the results between groups and across age for all the remaining perceptive tests are summarized in Figure 1. Significantly better performances of musicians were found on the BAT, MDT, and MPT. Significant differences were also found between year groups on the same cognitive and perceptive tests; moreover, an additional significant difference between groups was found for the RAT and between year groups for the EDT. Based on these ANOVA models, *post hoc* multiple comparisons using Bonferroni correction revealed significant differences between groups for all the tests except for EDT. Significant differences for grades were also found for all the tests except for JAJ and EDT. Significant differences for grades x group interaction were found throughout all the variables except for EDT.

**FIGURE 2:**
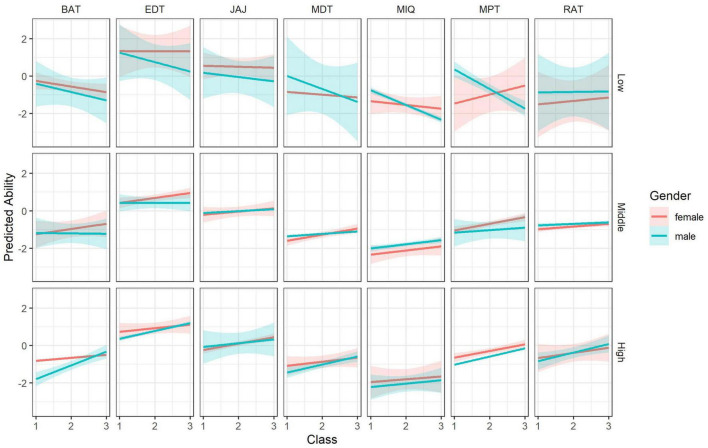
Predicted ability values for the seven performance tests by class (X-axis) and gender (red = females, blue = males) for the three levels of General Music Sophistication. BAT, Beat Alignment Test; EDT, Emotion Discrimination Test; JAJ, Jack&Jill Working Memory Test; MDT, Melody Discrimination Test; MIQ, Matrix Reasoning; MPT, Mistuning Perception Test; RAT, Rhythm Ability Test.

As significant differences were found in the starting *t*-test for the General Music Sophistication Index in 1st grade, a series of seven ANCOVAs were also employed treating the GOLD-MSI General Index score as a covariate. Significant differences between musicians and non-musicians remained significant both for JAJ (df = 1; *F* = 6.008; *p* = 0.015; ηp^2^ = 0.022) and MIQ (df = 1; *F* = 9.735; *p* = 0.002; ηp^2^ = 0.035) and as well as for year groups. Regarding the perceptive tests, the results for BAT and MPT barely changed after controlling for GMSI and they remained significant for the music groups. For the MDT results remained significant for both groups and years, while for the RAT no changes were found. Gender was also added as a variable, and the results are summarized in Table 7. Significant differences were found for EDT and MPT in favor of females. Predicted abilities showing the predictions of the final model with gender as covariate are summarized in Figure 2.

**TABLE 7:** ANCOVAs results for gender after Bonferroni correction and controlling for general music sophistication.

Measure	Test	df	*t*	*p*	p_bonferroni_
					
Visuo-spatial WM	JAJ	263	−0.18	0.857	0.857
Fluid Intelligence	MIQ	263	−0.47	0.637	0.637
Beat perception	CA-BAT	263	1.81	0.071	0.071
Melody Discrimination	MDT	263	0.11	0.908	0.908
Mistuning Perception	MPT	260	2.27	0.024*	0.024*
Emotion Discrimination	EDT	264	2.29	0.023*	0.023*
Rhythm Ability	RAT	262	−0.93	0.353	0.353

JAJ, Jack&Jill Working Memory Test; MIQ, matrix reasoning; CA-BAT, Computerized Adaptive Beat Alignment Test; MDT, Melody Discrimination Test; MPT, Mistuning Perception Test; EDT, Emotion Discrimination Test; RAT, Rhythm Ability Test.

Bold values represent the significant values.

Finally, *post hoc* multiple comparisons using Bonferroni correction based on the ANCOVA models showed significant differences between groups for all the tests except for EDT (Table 8); across age, significant differences were also found for all the tests but not for JAJ and EDT. Several significant interaction effects grades x groups were found except for EDT. In both *post hoc* analysis significant interaction effects were mostly found for comparisons between 1st Grade non-musicians and 2nd/3rd Grades musicians. The only exception was found for MDT where significant grades x groups interactions were found between 2nd Grade non-musicians - 3rd Grade musicians and 3rd Grade musicians - 3rd Grade non-musicians.

**TABLE 8:** ANCOVA’s *post hoc* comparison (Bonferroni correction) for grade, group and grade × group interaction.

Measure	Test	df	*t*	*p*	p_bonferroni_
					
Visuo-spatial WM	JAJ				
Grade		–	–	–	–
Group		263	2.78	0.012*	0.012*
Grade × Group Grade 1, NM - Grade 3, M		263	−3.71	<0.001*	0.004*
Fluid intelligence	MIQ				
Grade 1–3		263	−263	0.009*	0.027*
Group		263	3.32	0.001*	0.001*
Grade × Group Grade 1, NM - Grade 2, M		263	−3.18	0.002*	0.024*
Grade 1, NM - Grade 3, M		263	−4.10	<0.001*	<0.001*
Beat perception	CA-BAT				
Grade 1–2		263	−2.97	0.003*	0.010*
Group		263	2.19	0.029*	0.029*
Grade × Group Grade 1, NM - Grade 2, M		263	−3.80	<0.001*	0.003*
Grade 1, NM - Grade 3, M		263	−3.12	0.002*	0.030*
Melody discrimination	MDT				
Grade 1–3		263	−2.71	0.007*	0.021*
Group		263	3.55	<0.001*	<0.001*
Grade × Group Grade 1, NM - Grade 3, M		263	−4.37	<0.001*	<0.001*
Grade 1, M - Grade 3, NM		263	−2.98	0.003*	0.047*
Grade 2, NM - Grade 3, M		263	−3.25	0.001*	0.019*
Grade 3, M - Grade 3, NM		263	3.16	0.002*	0.026*
Mistuning Perception	MPT				
xgGrade 1–3		260	−3.31	0.001*	0.003*
Group		260	2.15	0.032*	0.032*
Grade × Group Grade 1, NM - Grade 2, M		260	−3.00	0.003*	0.044*
Grade 1, NM - Grade 3, M		260	−3.74	<0.001**	0.003*
Emotion discrimination	EDT				
Grade		–	–	–	–
Group		–	–	–	–
Grade*Group		–	–	–	–
Rhythm Ability	RAT				
Grade 1–3		262	−2.47	0.014*	0.043*
Group		262	3.57	<0.001*	<0.001*
Grade × Group Grade 1, NM - Grade 3, M		262	−4.45	<0.001*	<0.001*

(1) M, musicians; NM, nonmusicians.

(2) Comparisons are based on estimated marginal means.

(3) Only significant effects obtained after Bonferroni correction were included in the table.

(4) Df value is lower as a filter was applied to SEM values < 1.5 which removed the score of 9 participants; for MPT and RAT some participants are missing.

(5) JAJ, Jack&Jill Working Memory Test; MIQ, matrix reasoning; CA-BAT, Computerized Adaptive Beat Alignment Test; MDT, Melody Discrimination Test; MPT, Mistuning Perception Test; EDT, Emotion Discrimination Test; RAT, Rhythm Ability Test.

*represent the significant values.

## Discussion

The present study was carried out with the aim of evaluating the effects of music training on audiovisual WM and fluid intelligence in preadolescence. In particular, the focus was on instrumental training carried out in musical middle schools. For this purpose, a series of cognitive and perceptual tests were employed to check whether there is any difference between the two groups of musicians and non-musicians and over the years. Fluid intelligence, visuo-spatial WM, beat perception, melodic discrimination, mistuning perception, rhythmic ability, emotional discrimination and general music sophistication were assessed.

First of all, from analyzing the descriptive data for the JAJ test we found a lower percentage of below-zero results for the musicians, which seems significant considering that the participants belonging to the experimental group outnumber the participants of the control group. Furthermore, differences between the two groups in music perceptual tests could be traced back to pre-existing dissimilarities. In fact, a significant difference emerged in general music sophistication between the musical and non-musical group in the 1st grade, probably due to the fact that children in the musical middle schools are selected through aptitude tests to be enrolled in the music curriculum, and therefore the general musical abilities appear to be more enhanced in the musical group from the beginning.

Taken together, the results allow us to reject the null hypotheses, which predicts the absence of significant differences between the musical and non-musical group and over the years; in fact, significant results in musicians and for years, with a considerable variance between groups, were found. We would state that at least two of the three alternative hypotheses of the present study can be accepted: in one of them, we predicted the association between music training and superior audiovisual WM and fluid intelligence and, actually, the group of musicians showed a better performance compared to non-musicians in both domains. The present results converge with prior studies where music training was associated with superior cognitive abilities (Schellenberg, 2006, 2011b; Degé et al., 2011; Slevc et al., 2016; Baker et al., 2018; Meyer et al., 2020; Porflitt and Rosas, 2022).

In line with the first hypothesis, results also showed a significantly better performance by musicians in most of the perceptual tests. Since the music perceptual LongGold tests contain memory retention among the cognitive processes evaluated, similar results in these tests suggest that music training is associated with auditory memory, although, as already mentioned, this may possibly be due to pre-existing differences and selection. In fact, in previous studies, connections with potentially pre-existing auditory cognitive abilities that may help to explain the discrepancies between musicians and non-musicians were discussed (Mankel and Bidelman, 2018; Bidelman and Mankel, 2019). Note that significant differences remained even after controlling for general music sophistication for all the perceptive tests except for EDT, suggesting a convergence with the studies that found correlations between music training and auditory WM due to the near transfer effect (Kraus et al., 2012; Zuk et al., 2014; Slater and Kraus, 2016; Slevc et al., 2016; Alain et al., 2018; Nie et al., 2021).

Furthermore, the second alternative hypothesis that predicted a better performance from older groups due to maturation can partially be accepted; having found clear differences between the means of the three age groups suggests a maturation effect. However, the relative measurement error for the younger age group provides some uncertainty around these results. The high measurement error might be due to some younger students not obtaining a very good understanding of the task, but this can be verified with subsequent longitudinal data. Something similar was found for the perceptive tests: the tendency to improve the performance over the years seems to be an effect of the general maturation. These results converge with those of Rauscher and Hinton (2011) where children showed improvements in the third year of study, although in that case it was a longitudinal analysis unlike the present study. Here, an exception is represented by JAJ and EDT. For the MIQ results, on the other hand, growth is rather gradual over the years.

In the specific case of gender, previous studies suggested that boys may experience a window of increased sensitivity to risk-taking throughout adolescence that is broader in magnitude and longer in duration than females (Bennett et al., 2005; Shulman et al., 2015). This could be due to a difference in maturation between the two genders that would lead girls to show a better self-control and, by consequence, a better academic achievement (Matthews et al., 2009; Duckworth et al., 2019). Particularly, for WM a slight tendency in favor of females was found as well because of the existence of gender-specific strategies produced by different neurodevelopment trajectories, more specifically by hemispheric differences in the neural substrate. In fact, females tend to perform more accurately at the cost of longer reaction times (Grissom and Reyes, 2019). Although, in the specific domain of visuo-spatial WM, males seem to have an advantage (Voyer et al., 2017). In spite of this evidence suggesting differences between genders in cognitive maturation, no significant differences were found for the visuo-spatial WM nor for intelligence. However, significant results were found for MPT and EDT. In addition, the latter results clearly indicate a higher emotional maturation of females at this stage of development.

Finally, for the third and last hypothesis the question is more controversial: comparing the estimated marginal means after controlling for music sophistication showed a difference from the start between groups, both for MIQ and JAJ as well as the perceptive tests, and also a difference between 1st and 3rd grades consistently with a better performance by the group of musicians. Moreover, after Bonferroni correction some significant interaction effects were found for grades and groups. However, this would be not a definite proof yet of the positive effects of music training. Certainly, the starting difference between groups is clear-cut, so the contribution of other different factors to these results cannot be excluded, as the developmental trajectories of musical experience may be influenced by individual variations in musical ability, personality and cognitive capacity as well as environmental factors, such as socioeconomic background (Vincenzi et al., 2022). However, it can certainly result in a concrete hypothesis for further studies where further information may be provided by exploring these variables.

Similarly to the study carried out by Carioti et al. (2019), in the present study we aimed to demonstrate how the positive association between music training and better performance in visuo-spatial WM and fluid intelligence increase with increasing years of training, despite individual differences and other variables. In addition, we found improvements in auditory memory. But differently from the Carioti study, the present study is a cross-sectional analysis across the different ages and the three grades of students of the Italian musical middle schools that would serve as a hypothesis for future longitudinal analyses. Another difference and a strength of our study lies in having tested a larger sample size, recruited in three different schools located in three different parts of Bari’s metropolitan area and characterized by different social contexts. Moreover, a further difference to the Carioti study consists in a clearer distinction in terms of musical experience between the groups within the sample to reduce the risk of spuriousness due to the presence of music-skilled subjects in the control group.

In sum, the contributions of the present study certainly allow to add evidence to previous studies where music training had been correlated to better performance in visuo-spatial and auditory WM (Pallesen et al., 2010; George and Coch, 2011; Schulze et al., 2011a; Suárez et al., 2016; Anaya et al., 2017). As regards the specific domain of visuo-spatial WM, our results highlight the connection between instrumental training and implicit sequence learning. Similarly, to the study by Anaya et al. (2017), we found better performance by the group of musicians in the JAJ; in their study, participants were shown a sequence of black squares that were individually illuminated to form a sequence, and they were asked to reproduce it. The sequence was scored as correct if the participant was able to reproduce the entire sequence without error. In our study, one of the two tasks of the JAJ concerns precisely remembering increasingly long sequences of ball positions according to Jack’s rotation. Therefore, the JAJ task deals with memory encoding, which is one of the critical processes, together with storage and retrieval, involved in sequential memory (Liang et al., 2020), so, it is plausible find a better performance in musicians engaged in formal music training with this type of task.

It is certain that playing an instrument requires a lot of discipline, effort and patience. It requires also rote work and many hours of practicing and involving procedural memory and sequence processing. Sequences generally seem to be learned implicitly, in which case there is no relationship with WM. On the contrary, sequence learning tasks show WM differences if the learning is explicit or purposeful, as WM actively learns to direct attentional focus and cognitive control (Janacsek and Nemeth, 2013). It therefore seems conceivable that music training, together with other influencing factors, is able to affect sequence learning. Our findings seem to match the findings by Pau et al. (2013), who discovered a relationship between musicians’ functional brain alterations and memorizing visual sequences; however, such outcome may be a reflection of music students’ background in combination with their formal education, activities and years spent playing an instrument that are not present here.

Furthermore, we found a wider gap in 3rd grade scores in favor of musicians compared to the remaining school grades, but no Grade × Group interaction was found except for melody discrimination. Thus, it is not an inferential result and further studies are needed in order to enrich the literature on the possible effects of reiterated music training. Swaminathan et al. (2017) showed that music abilities, rather than the amount of music training, predicted far transfer effects in a sample of adults. This finding supports the idea that the correlation between far transfer effects and engagement in music is mediated by the level of music skills rather than the duration.

On the other hand, Criscuolo et al. (2019) showed significant positive relationships between WM and the duration of musical practice, even after controlling for intelligence and background variables, such as personality traits. It may be that the extent of the training influences the persistence of the effect after termination of the training. According to these studies it may be assumed that music training would be able to prepare a basis for a range of skills and thus promote cognitive development (Miendlarzewska and Trost, 2014). In addition, music training over time can be made progressively more challenging, and this is a unique strength to consider in future research when advocating for music training as a cognitive growth intervention (Cooper, 2020).

Overall, the contributions of this study concern the use of new instruments suitable for preadolescents to assess these domains for the first time with Italian children; the exploration of a new environment of study together with curricular instrumental training and the providing of new evidence to preadolescents, cognitive abilities and music training literature (Schellenberg, 2011a; Corrigall et al., 2013; Bergman Nutley et al., 2014; Putkinen et al., 2015; Carioti et al., 2019). Finally, our study provided new evidence for gender differences regarding the improvement of auditory skills in favor of females, with a particularly strong effect for the musical emotion recognition task.

In sum, music middle schools can be considered a resource for preadolescent education, a delicate stage of development in which it is necessary to offer the child the possibility of an overall maturation and to carry out group activities for a good transition to adulthood. Hence, in this sense, music middle schools certainly constitute an excellent public and free resource for the community. Even if the present results are not sufficient to direct research on WM and cognitive characteristics related to auditory stimulation and middle schools’ music training toward solid evidence, the promising lines of this study may be considered surely encouraging.

## Limitations and future research

Despite the contributions to the field, the present study is subject to several limitations. First of all, the cross-sectional setting does not allow to comment strongly on the influence of music training, since without longitudinal data it is not possible to establish a true cause and effect relationship; in fact, longitudinal experimental investigations are the most reliable proof that reading and musical experience are causally related ability (Tierney and Kraus, 2013).

Thus, the present study could be considered a cross-sectional examination of variations in musical ability and visuo-spatial WM comparing people with and without music training in three distinct age groups. However, cross-sectional studies can estimate prevalence of outcome of interest and are useful for the generation of hypotheses (Levin, 2006). Furthermore, as previously mentioned, the MiddleMusic project which this study is part of is still under progress and, throughout the following years, longitudinal data from the same children will be gathered.

Ideally, measurements should be conducted before training, immediately following training, and after training to see if any training-related advantages persists. Moreover, the control group should be active to leave out the possibility that trained children are performing better than control children merely because they are getting a treatment that the other group is not (Tierney and Kraus, 2013). In this regard, another important limitation of this study consists in the lack of an active control group together with the pre-existing differences between the two groups, as other factors such as receiving teachers’ increased attention, more interaction with peers or more time spent at school may influence results; although, it might be logistically challenging to find an active control training program that matches music training in the same level of intensity and enthusiasm. Additionally, even if several extra-curricular projects addressed to all students are put on throughout the school year, the specific activities of our sample were not investigated. As regards pre-existing differences, they may determine the desire or ability that led some of the children to study music and, by consequence, the differences between groups; in this regard, the initial choice of school curriculum alone seems not to be sufficient to isolate the influence of other potential factors. In fact, a future musician may decide to enroll in an educational program with intensive music training due to his predispositions, making it difficult to determine whether the cognitive advantage seen in musically trained children is a genuine effect of the training, whether it is a specific one, or whether it is a spurious effect. According to the latter approach, Schellenberg’s argument that music training is better suited for analyzing pre-existing differences in brain and cognitive development than plasticity brought on by training would be confirmed (Schellenberg, 2011a).

The risk of the high impact of pre-existing differences on differences between groups occurs especially when children are not randomly allocated to either a music training group or a control group. Since the current study is quasi-experimental research, random assignment is another crucial topic to deepen. According to Barrett et al. (2013), it can be difficult to conduct a study on the effects of music training with randomization. As a result, there is frequently a compromise between the ecological validity of musical education and the degree to which study designs can satisfy these requirements. The results of such investigations will be challenging to adapt to actual music learning environments, but using the experimenter’s training programs can enable more rigorously controlled trials (Barrett et al., 2013). On the other hand, researchers might increase the relevance of their findings to educators at the expense of certain study design restrictions by looking at current programs that have been shown effective in teaching adults and children musical abilities (Fasano et al., 2020). The method used to establish group equivalency is crucial when proper randomization is not realistically feasible.

Also, due to lack of a second-level interaction, here and in the previous study by Carioti et al. (2019) it might be concluded that 1 year of intensive music training alone is not sufficient to strongly affect cognitive maturation. However, cognitive and emotional abilities and mutual acceptance among children can be further benefited, regardless of the relatively advanced stage of children’s emotional maturation, although further research is required on the topic. Hence, overall, our findings allow us to join the chorus of other researchers in suggesting that primary school could be exploited as a privileged environment and should include music training as a significant tool to aid cognitive maturation (Rauscher and Hinton, 2011; Hallam, 2015; Altenmüller, 2016; Carioti et al., 2019; Peretz, 2019; Kraus and White-Schwoch, 2020). In fact, starting music training in primary school and persevering with it also during pre-adolescence might more likely produce significant results on cognitive maturation.

These findings stand upon neural basis; as already mentioned, increasing neuroscientific research demonstrates its positive effects on brain development. For example, musical expertise is associated with the efficiency of a right-lateralized frontoparietal network activated by the EF tasks, meaning that musically trained children are able to engage neural resources that are inaccessible to untrained children, perhaps because of earlier maturation, stronger task engagement, or the use of different cognitive strategies (Putkinen and Saarikivi, 2018).

Again, the question is if these effects are the product of intensive music training rather than of other factors, such as pre-existing differences or biological traits of musicality. The anatomical evidence reveals that musicians have specific brain areas that appear different compared to the starting age of music training; (e.g., a study with functional magnetic resonance imaging identified the left superior temporal gyrus as the region that is linked with music training, in terms of cumulative practice hours (Ellis et al., 2013). Diffusion tensor imaging studies, on the other hand, show greater structural connectivity in other areas (Schlaug, 2015; Møller et al., 2021; Criscuolo et al., 2022). A further longitudinal study with pre-adolescents showed an improvement in some perceptual and cognitive abilities after a 6-month individual instrumental training carried out at a public music school and also a neuroplastic change of the frontotemporal networks of brain connectivity, measured with neuroimaging techniques (Cantou et al., 2018; Brattico, 2019; Fasano et al., 2020). Then, according to these studies, it is reasonable to assume that music training can induce brain changes not attributable to pre-existing differences or biological traits.

Recent research has also shown that the child’s agreeableness and the parents’ openness to new experiences are the best indicators of how music lessons would affect the youngster (Corrigall and Schellenberg, 2015). In this respect, individual variables, such as personality or socioeconomic status that could affect outcomes, are important to explore in further studies; as these factors have not been analyzed in the present study, this correlation should be verified by evaluating the personality types of the parents and the children. Mostly, to contrast the limitations of this study, more longitudinal and random assigned trials on pre-adolescent musicians are needed when feasible. In addition, the presence of the active control condition is prominent and, importantly, both experimental and active control groups should be tested for far transfer (Bigand and Tillmann, 2022). This would allow to make equal and fair comparisons and to have a clearer view on the possible causal effects of music training; therefore, it is essential to move in this direction for future research.

## Data availability statement

The dataset analyzed for this study can be found in the Zenodo digital repository at doi: 10.5281/zenodo.6958523.

## Ethics statement

The studies involving human participants were reviewed and approved the Ethics Committee, Department of Education, Psychology, Communication University of Bari “Aldo Moro”, Italy. Written informed consent to participate in this study was provided by the participants’ legal guardian/next of kin.

## Author contributions

ML: design, coordination, recruitment, data acquisition, data management, data analysis and interpretation, and manuscript writing. EB: conceptualization, design, recruitment, data analysis and interpretation, and manuscript revision. DM: design, data management, data analysis and interpretation, and manuscript revision. KF: design, data management, figures preparation, and manuscript revision. BM: data acquisition, data interpretation, and manuscript revision. PV: design, funding, and manuscript revision. RC: design and manuscript revision. All authors contributed to the study and to the final revision of the manuscript.

## Footnotes

1
www.miur.gov.it

2
https://ec.europa.eu/

3
https://www.istat.it/

4
https://www.istruzione.it/pon/index.html

5
https://longgold.org/

6
https://github.com/klausfrieler/JAJ
